# Sympathetic Activation and Arrhythmogenesis after Myocardial Infarction: Where Do We Stand?

**DOI:** 10.3390/jcdd8050057

**Published:** 2021-05-15

**Authors:** Konstantinos C. Zekios, Eleni-Taxiarchia Mouchtouri, Panagiotis Lekkas, Dimitrios N. Nikas, Theofilos M. Kolettis

**Affiliations:** 1 1st Department of Cardiology, University Hospital of Ioannina, 1 St. Niarxou Avenue, 45500 Ioannina, Greece; zekioskostas@gmail.com (K.C.Z.); dimitrios.nikas@gmail.com (D.N.N.); 2 Department of Cardiology, University of Ioannina, 1 St. Niarxou Avenue, 45500 Ioannina, Greece; elenimouch@gmail.com; 3 Cardiovascular Research Institute, 1 St. Niarxou Avenue, 45500 Ioannina, Greece; panlek1981@gmail.com

**Keywords:** myocardial infarction, ventricular tachyarrhythmias, sympathetic activation

## Abstract

Myocardial infarction often leads to progressive structural and electrophysiologic remodeling of the left ventricle. Despite the widespread use of β-adrenergic blockade and implantable defibrillators, morbidity and mortality from chronic-phase ventricular tachyarrhythmias remains high, calling for further investigation on the underlying pathophysiology. Histological and functional studies have demonstrated extensive alterations of sympathetic nerve endings at the peri-infarct area and flow-innervation mismatches that create a highly arrhythmogenic milieu. Such accumulated evidence, along with the previously well-documented autonomic dysfunction as an important contributing factor, has stirred intense research interest for pharmacologic and non-pharmacologic neuromodulation in post-infarction heart failure. In this regard, aldosterone inhibitors, sacubitril/valsartan and sodium-glucose cotransporter type 2 inhibitors have shown antiarrhythmic effects. Non-pharmacologic modalities, currently tested in pre-clinical and clinical trials, include transcutaneous vagal stimulation, stellate ganglion modulation and renal sympathetic denervation. In this review, we provide insights on the pathophysiology of ventricular arrhythmogenesis post-myocardial infarction, focusing on sympathetic activation.

## 1. Introduction

**Figure 1:**
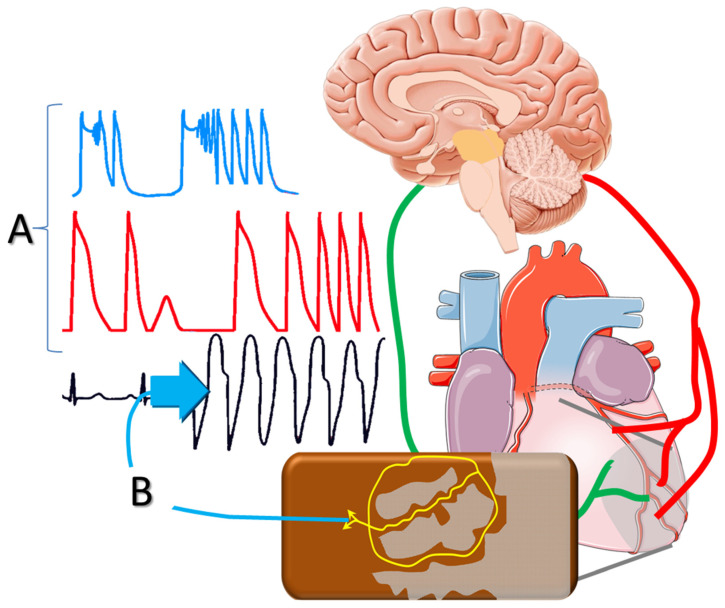
Sympathetic activation post-infarction triggers delayed afterdepolarizations and polymorphic ventricular tachycardia; and (**A**) facilitates reentrant mechanisms via (**B**) altering the conduction properties of the peri-infarct tissue.

Myocardial infarction (MI) often leads to substantial loss of contractile tissue, despite prompt revascularization in the acute phase. Due to the high incidence of coronary artery disease, progressive left ventricular (LV) dilatation and dysfunction post-MI constitutes the most frequent cause of heart failure, which is associated with high morbidity and mortality. Recent evidence demonstrates that heart failure post-MI remains a major health-related problem worldwide, diagnosed in 20–30% of patients one year after the acute event, with the rates steadily rising thereafter [1]. In addition to disabling symptoms and frequent hospitalizations, heart failure is also associated with high incidence of ventricular tachyarrhythmias (VTs), often heralding sudden cardiac death (SCD). The most effective antiarrhythmic strategy is hitherto offered by implantable cardioverter-defibrillators (ICDs), which promptly terminate VTs and prolong survival in selected post-MI populations [2]. Combined with cardiac resynchronization, device-therapy can also ameliorate LV asynchrony and, thereby, improve overall LV performance [3]. However, the value of ICD implantation is hampered by major limitations in current risk stratification algorithms that often fail to identify patients at risk for SCD [4]. As a result, there is an utter need for better understanding of the mechanisms underlying arrhythmogenesis in the post-MI setting, with a view towards expanding the present pharmacologic and non-pharmacologic armamentarium. In this review, we examine pathophysiologic aspects of ventricular arrhythmogenesis in the setting of healed MI. We also discuss the role of substrate formation post-MI and the effects of autonomic imbalance, placing emphasis on sympathetic activation (Figure 1).

## 2. Myocardial Salvage

The extent of myocardial necrosis after acute coronary occlusion and the resultant LV dilatation and dysfunction are the most important predictors of long-term outcome. In this regard, the widespread use of acute reperfusion strategies in recent years has had major impact on the incidence of SCD post-MI [5]. Thrombolysis, and mainly, percutaneous coronary interventions decrease infarct size and transmurality, thereby, ameliorating the substrate for chronic-phase VTs. Therefore, acute MI patients, presenting early in tertiary hospitals, seem to have excellent prognosis during long-term follow-up [6]. However, a substantial proportion of patients with acute MI present too late for myocardial tissue salvage by revascularization; this subgroup, currently estimated at the range of 20%, is characterized by high short- and long-term complication rates [7].

## 3. Ventricular Remodeling Post-MI

Myocardial necrosis alters loading conditions and triggers a cascade of events, frequently referred to as LV remodeling [8]. Commencing during the early hours after acute coronary occlusion, myocardial loss increases local wall stress in the infarcted area, causing expansion of the border zone [9]. This course is closely intertwined with infarct healing, a dynamic response that initiates a reparative process, leading to collagen scar formation [10]. Structural remodeling is histologically characterized by fibrosis, which is evident not only in the tissue within and adjacent to the infarct, but also in remote myocardial areas. In the presence of fibrotic tissue, areas of slow conduction provide the substrate for the formation of reentrant circuits. Importantly, such structural LV remodeling may continue for weeks or months after the acute event and eventually presents clinically as overt heart failure [8]. This process is accompanied by altered electrophysiologic properties, also known as electrical remodeling, evident in areas adjacent to the infarct scar, as well as in the non-infarcted myocardium [11]. As part of this process, abnormal expression and distribution of connexins alter gap junction function and further compromise electrical conduction [12]. Impaired calcium signaling is a hallmark finding in heart failure at the cellular level, with post-MI remodeling associated with distinct features describing the spatial location and function of L-type calcium channels [13]. Therefore, calcium sparks, manifesting as early or delayed afterdepolarizations, constitute an additional arrhythmogenic mechanism in post-MI heart failure [14].

## 4. Increased Sympathetic Drive

The autonomic nervous system consists of a complex set of neurons that regulate different systems, aiming at maintaining homeostasis by adapting to changes of the external and internal environment. During emotional stress or exercise, the sympathetic nervous system increases heart rate, conduction velocity and contractility, allowing for the increased metabolic demand. Increased sympathetic drive also occurs as an adaptive response in heart failure, enhancing the contractility of the healthy myocardium. However, this action is associated with several maladaptive mechanisms that increase arrhythmogenesis in the long-term, a process that has been attracting major research interest for decades.

Chronically elevated sympathetic drive affects a number of ionic currents (referred to as ionic remodeling), resulting in global alteration of LV electrophysiologic properties [15]. For instance, norepinephrine reduces the inward rectifier potassium current, increases resting membrane potential and enhances abnormal automaticity, an action mediated via β-adrenergic receptors. In the presence of a substrate that is invariably located in the peri-infarct tissue, extrasystolic activity may facilitate the onset of monomorphic VTs [16]. Re-entrant mechanisms are also facilitated by chronic β-receptor stimulation via topographic and functional alterations of connexins [17]. Moreover, norepinephrine shortens the duration of the ventricular action potential and elicits delayed afterdepolarizations at high heart rates, thereby, triggering polymorphic VTs that can degenerate into ventricular fibrillation. Last, sympathetic activation affects the restitution features of the ventricular myocardium and alters its refractory period. The latter effects differ, depending on the baseline regional electrophysiologic properties, with refractory period shortening in normal myocardium, as opposed to prolongation in the border zone, surrounding the infarcted tissue. As a result, sympathetic activation can markedly enhance repolarization dispersion in the peri-infarct zone and sets the stage for reentrant mechanisms [18].

## 5. Antiarrhythmic Effects of β-Blockade

The widespread use of β-blockers has been a major advance in the treatment of post-MI patients during the past decades. These agents exert anti-ischemic actions by reducing myocardial metabolic demand and by prolonging diastolic perfusion time secondary to their actions on the sinus node. Moreover, β-blockade obviates action potential changes and increases the threshold for ventricular fibrillation, counterbalancing the pro-fibrillatory effects of myocardial ischemia or sympathetic activation [19]. Based on these anti-ischemic and anti-arrhythmic properties, β-blockade has been consistently shown to lower SCD rates in post-MI patients. For example, a meta-analysis of 30 trials (totaling almost 25,000 patients) examining the effects of β-blockade post-MI, showed a 31% reduction in SCD rates over a mean follow-up period of approximately one year, yielding a corresponding benefit in cardiovascular and all-cause mortality [20]. However, the antiarrhythmic potential of β-blockade seems to decline over time [21], due to progressive structural and electrophysiologic alterations in the substrate [22]. Based on the above considerations, the need for further research towards lowering SCD rates post-MI remains a pressing issue of major clinical importance.

## 6. Local Versus Circulating Epinephrine

Sympathetic responses entail two major components, namely increased circulating catecholamines from the adrenal medulla, as well as local norepinephrine release from sympathetic nerve endings, elicited by central activation. The pathophysiologic and clinical significance of such distinction has been largely overlooked, a notion likely attributed to the well-studied downstream-effects of β-blockade. Nevertheless, several pieces of recent data highlight the implications of such disparate electrophysiologic effects of sympathetic activation [23]. For example, (left, right, or bilateral) stellate ganglia stimulation produced distinct patterns of repolarization sequence in the normal porcine heart, assessed by analysis of epicardial and endocardial electrograms [24]. These recordings revealed marked dispersion of repolarization, which was absent when the effects of circulating norepinephrine were examined. Therefore, current evidence suggests that the dispersion of repolarization induced by local sympathetic activation in the peri-infarct area is highly arrhythmogenic, contrasting the more uniform alterations, induced by circulating catecholamines.

## 7. Flow–Innervation Mismatch in Healed MI

In the process of infarct healing, the area of abnormal myocardial blood flow in the peri-infarct tissue is often surrounded by larger areas, characterized by regional impairment of neuronal catecholamine uptake [25]. Such impairment consists of areas with diverse nerve density and increased neuronal excitability that predisposes to arrhythmogenesis [26].

**Figure 2:**
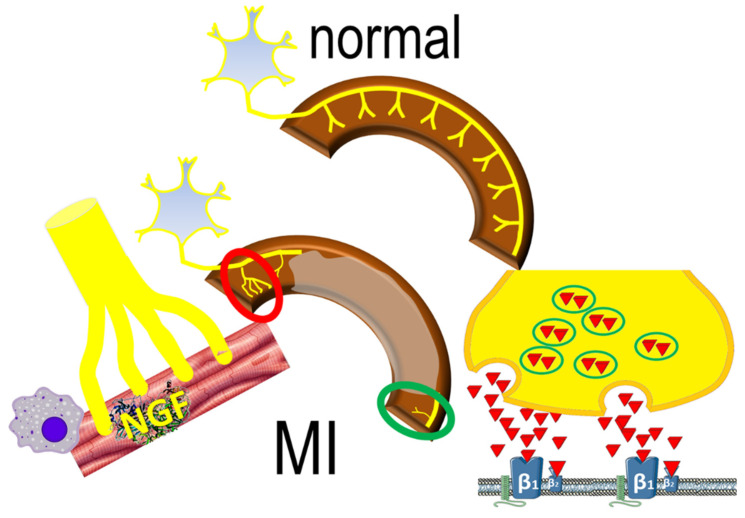
The normal sympathetic nerve distribution (upper panel) is replaced by sympathetic nerve remodeling post-infarct (lower panel), characterized by areas of increased (red circle) sympathetic nerve density, mediated by inflammatory responses; decreased (green circle) nerve density also occurs, characterized by norepinephrine overspill, activating β_1_ and β_2_ adrenergic receptors.

Areas of increased density of nerve-fibers adjacent to the infarcted have been reported in animal models of MI, stimulated by local elevations of nerve growth factor in response to ischemic injury [27]. Local sympathetic remodeling entails also areas of *decreased* denervation of viable myocardium around the infarct scar, as sympathetic nerve fibers are more susceptible to ischemia than cardiomyocytes. Interestingly, denervated myocardial regions display excessive responses to catecholamines (Figure 2), contributing to inhomogenous electrophysiologic milieu that favors the formation of reentrant circuits [28]. This apparently paradoxical effect may be the result of enhanced norepinephrine production and overspill in sympathetic nerve endings [29]. The downstream effects are potentiated by abnormal myocardial perfusion in the peri-infarct area that prevents effective blockade of β-adrenergic receptors.

## 8. The Role of Inflammation

Sympathetic nerve remodeling is a complex pathophysiologic process, of which the inflammatory response is thought to be an integral element. Signals from necrotic cells induce the expression of pro-inflammatory cytokines and chemokines that attract neutrophils and monocytes [30]. Activated macrophages play a key-role in sympathetic nerve growth post-MI, exerted mainly via the secretion of nerve growth factor. Inflammatory and autonomic responses may be further interrelated by afferent nerve activation via several cytokines, resulting in efferent modulation of healing responses [31]; the exact structural and biochemical processes are currently under investigation.

## 9. Central Actions of β-Blockade

The presence of β-adrenergic receptors in the central nervous system has been known for decades. They are located mainly in the hippocampus, the cerebellum, in thalamic nuclei and basal ganglia, as well as in the midbrain and cerebral cortex; low levels of β-receptors are also found in the white matter and hypothalamus [32]. Although the pathophysiologic role of central adrenergic mechanisms in hypertension are well-established [33], their importance in ventricular arrhythmogenesis in the presence of infarcted tissue remains unclear. This topic resurfaced after critical re-evaluation of several large post-MI clinical trials, arguing that antiarrhythmic efficacy should be considered established only for propranolol, timolol and metoprolol, i.e., agents penetrating the blood-brain barrier [34]; by contrast, no firm data could be substantiated for water-soluble agents [35]. Although the long-standing ‘lipophilicity hypothesis’ has not been verified, it paved the way for preclinical and clinical investigations focusing on the role of autonomic balance on cardiac electrophysiology [36].

## 10. Autonomic Modulation in Chronic Heart Failure

Based on the detrimental effects of central sympathetic activation, the centrally acting agent moxonidine was evaluated in a multicenter trial [37]. However, this trial had to be terminated early, because of excess morbidity and mortality in patients receiving active treatment, despite decreased plasma norepinephrine levels. The explanation for the adverse effects of moxonidine in congestive heart failure remains speculative, implicating complex pharmacologic effects of concurrent imidazoline receptor blockade [38]. Such incompletely understood drug-effects have shifted research interest towards non-pharmacologic autonomic modulation, with various exercise training regimens presenting as a safe and widely applicable approach [39]. Moreover, in addition to novel pharmaceutical approaches, there is growing interest in interventional procedures, aiming at modulating sympatho-vagal balance [40].

## 11. Non-Pharmacologic Strategies

**Figure 3:**
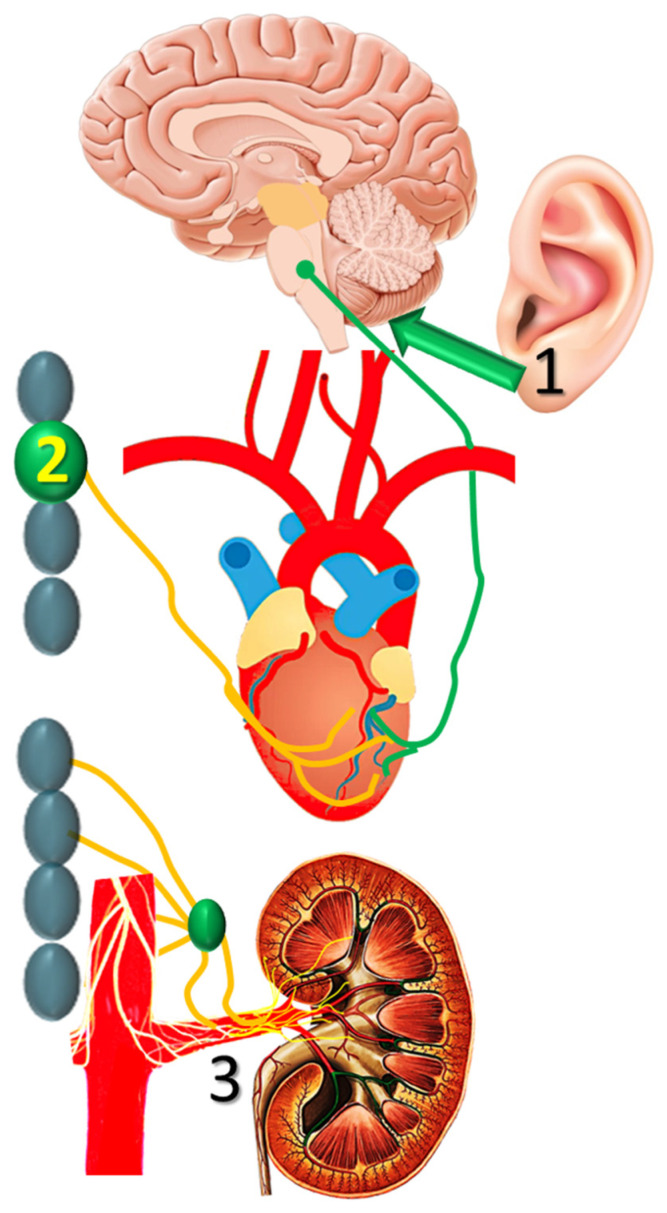
Transcutaneous vagal stimulation, (**1**) stellate ganglion modulation, and (**2**) renal sympathetic denervation are (**3**) currently investigated in nonpharmacologic autonomic modulation post-infarction.

A number of non-pharmacologic autonomic interventions are currently under intense investigation in the management of post-MI VTs [41]. Of these, transcutaneous vagal stimulation, stellate ganglion modulation and renal sympathetic denervation seem to have an advantage (Figure 3), as briefly discussed below.

### 11.1. Noninvasive Vagal Stimulation

Low-level tragus stimulation has been proposed as a noninvasive intervention for autonomic modulation, as the auricular branch of the vagal nerve is amenable to external stimulation. In dogs with healed MI, such intervention decreased the inducibility of VTs, an action partly attributed to reduced expression of nerve growth factor [42]. Preliminary clinical studies demonstrated favorable sympatho-vagal alteration, and larger scale clinical trials are expected to shed light on the efficacy of noninvasive vagal stimulation in suppressing post-MI VTs.

### 11.2. Stellate Ganglion Ablation

Modification of cardiac sympathetic nerve activity through left or bilateral stellate ganglion ablation has been used as a last resort for refractory ventricular arrhythmias, mostly in patients with implanted ICDs presenting with electrical storm. Long-term follow-up from such series indicates sustained favorable results after bilateral stellate ablation, with respect to recurrence of VTs and mortality [43]. However, the investigation is ongoing, as this method is limited by side effects, such as Horner’s syndrome, abnormal sweating and chest pain.

### 11.3. Renal Sympathetic Denervation

Renal sympathetic denervation may modulate autonomic activity, independent of its effects on blood pressure, as shown by the reduction of spontaneous VTs in a porcine model of healed MI [44]. This procedure showed promise as an adjunctive to stellate ganglion modulation in a small series of patients with VTs [45], but the results of this retrospective study need to be confirmed in sham-controlled randomized studies.

## 12. Pharmacologic Approaches

In addition to β-blockade, three further treatments have shown efficacy in reducing the incidence of SCD, namely aldosterone blockade, scubitril/valsartan and sodium-glucose co-transporter (type 2) inhibitors. In these paradigms, the issue of ameliorating central sympathetic activation has been raised, strongly reinforcing previous considerations.

### 12.1. The Aldosterone Blockade Paradigm

Spironolactone [46] or eplerenone [47], both examined in large multicenter clinical trials, lowered total mortality over a medium-term follow-up. This benefit results from attenuated progression of heart failure, as well as from lower SCD rates. The possible mechanisms underlying the antiarrhythmic properties of aldosterone blockade are multiple, with the prevention of serious hypokalemia and inhibition of the well-described effects of aldosterone on myocardial fibrosis appearing as highly likely [48]. Interestingly, an additional mode of action has been brought forward, describing lower central sympathetic drive by inhibiting direct actions of aldosterone on the hypothalamic paraventricular nucleus [49]. This hypothesis is supported by clinical data, indicating more favorable autonomic function in patients with post-MI heart failure receiving spironolactone [50].

### 12.2. The Sacubitril/Valsartan Paradigm

The recent PARADIGM-HF trial examined the efficacy of angiotensin receptor/neprilysin inhibition in patients with heart failure and reduced ejection fraction [51]; compared to enalapril, mortality was lower after sacubitril/valsartan, an effect evident regarding both, progressive pump failure and SCD [51]. In addition to improving substrate properties, central sympathetic effects appear also likely, calling for further investigation. More specifically, as neprilysin cleaves several peptides, it has been hypothesized that their increased concentration resulting from neprilysin inhibition may have antiarrhythmic effects [52]. A candidate molecule is enkephalin, increased levels of which accompany angiotensin receptor/neprilysin inhibition. Such endogenous opioid peptides appear to mediate sympatholytic and vagotonic responses via the opioid-receptors and may thereby exert potent autonomic effects [53].

### 12.3. The Sodium-Glucose Co-Transporter Type 2 Inhibitors Paradigm

Sodium-glucose co-transporter type 2 inhibitors are glucose-lowering agents that promote glucosuria, independent of the action of insulin. Recent multicenter clinical trials reported reduced cardiovascular mortality in diabetic patients with LV dysfunction [54]; the mechanisms underlying this benefit are likely multifaceted, including metabolic, endocrine, hemodynamic, and biochemical effects. Interestingly, experimental [55] and clinical [56] studies indicate the interactions of these agents with sympathetic nerves that innervate the proximal tubules of the kidney, thereby, lowering sympathetic drive.

## 13. Conclusions

Prompt revascularization of the infarct-related coronary artery decreases the necrotic area. Unfortunately, treatment delays are frequent, particularly in elderly patients or diabetics; hence, post-MI heart failure remains a common clinical entity, characterized by evolving structural and electrophysiologic milieu. Despite β-blockade, the incidence of VTs and SCD are high, as current risk-stratification algorithms cannot accurately identify patients that benefit from ICDs.

Several pieces of evidence point towards a crucial role of sympathetic activation on arrhythmogenesis that accompanies progressive post-MI LV remodeling. Examining the complex pathophysiology, a broad spectrum of pre-clinical and clinical studies is currently under way, addressing various aspects of the underlying mechanisms. Non-pharmacologic therapies are intensely investigated, introducing neuromodulatory approaches for post-MI heart failure. Beyond β-blockade, direct pharmacologic approaches were initially disfavored after the disappointing results of moxonidine, but the robust data of SCD reduction by aldosterone antagonists, by sacubitril/valsartan or, more recently, by the sodium-glucose cotransporter type 2 inhibitors have broadened the therapeutic horizon. The pathophysiologic role of the autonomic nervous system is an intriguing topic, with promising clinical applications in the chronic phase of MI.
